# Changes in Structural and Thermodynamic Properties of Starch during Potato Tuber Dormancy

**DOI:** 10.3390/ijms24098397

**Published:** 2023-05-07

**Authors:** Lyubov A. Wasserman, Oksana O. Kolachevskaya, Alexey V. Krivandin, Anna G. Filatova, Oleg V. Gradov, Irina G. Plashchina, Georgy A. Romanov

**Affiliations:** 1 Emanuel Institute of Biochemical Physics RAS (IBCP RAS), Kosygina Str. 4, 119334 Moscow, Russia; lwasserma@mail.ru (L.A.W.); a.krivandin@sky.chph.ras.ru (A.V.K.); o.v.gradov@gmail.com (O.V.G.); igplashchina@yahoo.com (I.G.P.); 2 Timiryazev Institute of Plant Physiology RAS (IPP RAS), Botanicheskaya Str. 35, 127276 Moscow, Russia; vatrushbox@mail.ru; 3 Semenov Federal Research Center for Chemical Physics RAS (ICP RAS), Kosygina Str. 4, 119991 Moscow, Russia; filatovaanna1@mail.ru

**Keywords:** starch, *Solanum tuberosum*, tuber dormancy, transgenic potato, *AtPHYB* gene, granulometric analysis, thermodynamic parameters

## Abstract

The main reserve polysaccharide of plants—starch—is undoubtedly important for humans. One of the main sources of starch is the potato tuber, which is able to preserve starch for a long time during the so-called dormancy period. However, accumulated data show that this dormancy is only relative, which raises the question of the possibility of some kind of starch restructuring during dormancy periods. Here, the effect of long-term periods of tuber rest (at 2–4 °C) on main parameters of starches of potato tubers grown in vivo or in vitro were studied. Along with non-transgenic potatoes, *Arabidopsis phytochrome B* (*AtPHYB*) transformants were investigated. Distinct changes in starch micro and macro structures—an increase in proportion of amorphous lamellae and of large-sized and irregular-shaped granules, as well as shifts in thickness of the crystalline lamellae—were detected. The degree of such alterations, more pronounced in *AtPHYB*-transgenic tubers, increased with the longevity of tuber dormancy. By contrast, the polymorphic crystalline structure (B-type) of starch remained unchanged regardless of dormancy duration. Collectively, our data support the hypothesis that potato starch remains metabolically and structurally labile during the entire tuber life including the dormancy period. The revealed starch remodeling may be considered a process of tuber preadaptation to the upcoming sprouting stage.

## 1. Introduction

Starch is a main storage polysaccharide of green plants, and it is widespread in nature. This substance is a biopolymer consisting of two polysaccharides (amylose and amylopectin), and their structures and proportions determine physicochemical and functional properties of extracted starches and respective products. Due to their characteristics and natural renewability, starches of various botanical origin are widely used in many industries as thickeners and as stabilizing and gel-forming agents. Of particular interest are starches for food production and medicine [1,2].

Potato (*Solanum tuberosum* L.) is one of the most important natural producers of starch. Starch is extensively accumulated in growing potato tubers, serving as long-term storage for carbohydrates, and is remobilized at the time of tuber sprouting [3]. After tubers ripen and reach their final size, they undergo a dormancy period. This period lasts during the winter and serves to preserve the tubers from vegetative propagation under unfavorable conditions. During dormancy, tuber resistance against pathogen attacks increases, which corresponds to the need to preserve starch and protein reserves for future seedlings [4,5]. In general, tuber dormancy is an adaptive reaction of potato ontogenesis, ensuring the successful reproduction of the *Solanum tuberosum* species [3,6].

Dormancy is now considered a temporary halt of the growth of any plant structure containing a meristem [7]. For potato tubers, three categories of dormancy are distinguished depending on how growth is blocked. In the period of deep dormancy (endodormancy), the proliferation of meristem cells ceases under the influence of internal physiological factors. During forced dormancy, growth is blocked by unfavorable external conditions (ecodormancy) or by physiological causes external to the given meristem (paradormancy) [6,8].

At the end of the dormancy period, the tuber begins to sprout giving rise to new plants, which terminate the final phase of the vegetative life cycle of the potato. The parameters of deep dormancy of tubers, including dormancy duration, are persistent hereditary traits [6]. At the same time, it is possible to control periods of dormancy and sprouting of potato tubers by using phytohormones, primarily gibberellins and abscisic acid [3,6,9]. Thus, potato tubers in the dormancy period retain sensitivity and reactivity with respect to at least some phytohormones.

Potato tubers can also arise in vitro on single-node explants or isolated stolons, and they differ from tubers grown in vivo mainly in size. The advantage of in vitro tuberization is a synchronized and faster response of plants to standardized experimental conditions [10,11,12], as well as the possibility of varying these conditions over a wider range than when cultured in vivo. The relative activities of enzymes involved in carbohydrate metabolism, as well as starch content from in vivo and in vitro tubers, have been shown to be similar [13].

One of the pressing issues related to the tuber dormancy concerns starch behavior. At first glance, in resting tubers, kept in the dark at a temperature not exceeding 4 °C, starch formation de novo seems to be unlikely. Since growth processes in dormant tubers do not occur, starch consumption for remaining metabolic needs such as residual respiration should be greatly reduced. This may lead to an assumption that in the rest period of tubers their starch stays virtually inert and is reactivated only in the sprouting period, serving cell growth and division. On the other hand, studies of mobile carbohydrate contents as well as the activities of a number of enzymes/genes of carbohydrate metabolism, including those involved in starch formation and breakdown, have revealed small but significant activity during the rest period of the tubers and even during deep dormancy [14,15,16].

The latter has opened the possibility of impact on starch structure in the period of tuber rest, as well. However, apart from some extensive studies of enzymes/genes and metabolites involved in starch synthesis and breakdown in dormant potato tubers, only a few attempts have been made to directly study the structure of starch itself [17,18]. Therefore, the aim of this work was to check possible changes in the structural and thermodynamic properties of potato starches depending on the length of time the tubers were at rest. This study was conducted on starches extracted from tubers of non-transgenic or *Arabidopsis phytochrome B* (*AtPHYB*)-transformed potatoes, cultivated either in vivo or in vitro. Earlier, we showed that *AtPHYB* transformants exhibited distinct structural changes in starch compared to non-transgenic control [19]. The current study shows that tuber dormancy does not preclude structural changes in starch, which remains metabolically and structurally labile even under rather hard (cold, darkness, and limited humidity) storing conditions. We suggest that the revealed structural changes of the starch are a kind of preadaptation to the upcoming sprouting stage.

## 2. Results and Discussion

### 2.1. Starch-Related Enzymatic Activity in Dormant Tubers

**Figure 1:**
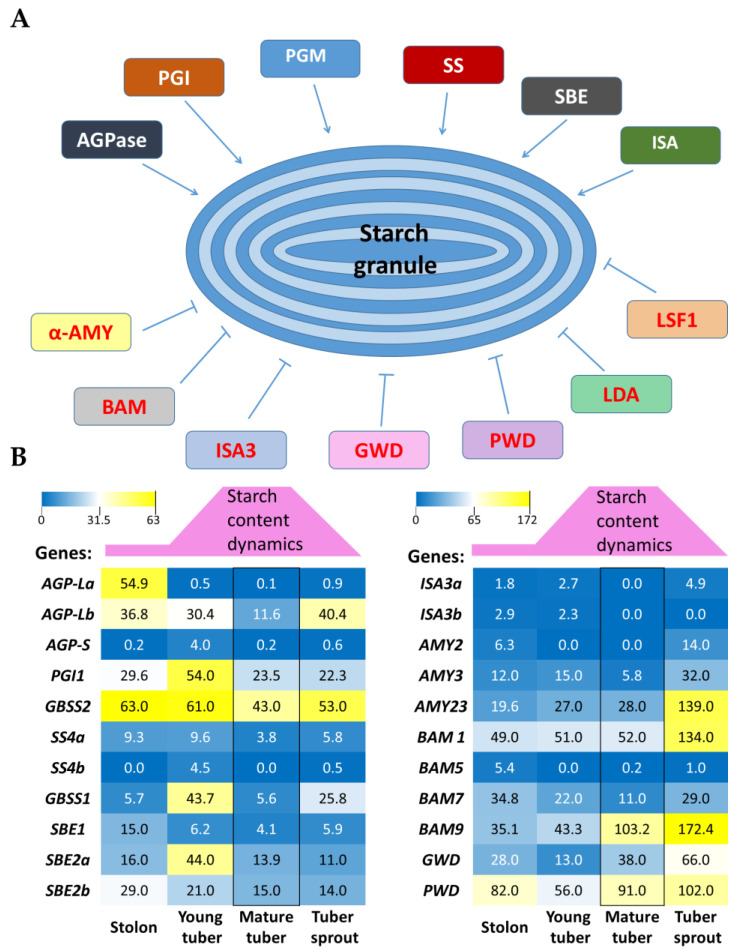
Starch biosynthesis and degradation during the process of tuber development. (**A**) Schematic illustration for the involvement of multiple enzymes in potato starch biosynthesis (upper line) and degradation (lower line). (**B**) Activity of the genes of starch biosynthesis (left) and degradation (right) at different stages of tuber development. Mature tuber stage is highlighted. Above the heatmap, conventional dynamics of starch content in a tuber is presented. Abbreviations are: *AGP-La,b–ADP-glucose pyrophosphorylase*, large subunit, a or b isomers; *AGP-S–ADP-glucose pyrophosphorylase*, small subunit; *PGI–phosphoglucoisomerase*; *SS–starch synthase*; *GBSS–granule-bound starch synthase*; *SBE1,2–starch-branching enzyme*, 1 or 2 isomers; *ISA3–isoamylase 3*; *AMY–α- amylase*; *BAM–β-amylase*; *GWD–glucan*, *water dikinase*; *PWD–phosphoglucan*, *water dikinase*; *PGM–phosphoglucomutase*; *LDA–limit dextrinase*; *LSF1-like Sex Four 1*.

Starch behavior in the cell depends on the activity of several enzymes, as well as availability of certain low molecular weight carbohydrates. Figure 1A demonstrates multiple enzymes, involved in starch biosynthesis and decay. The widely known accumulation of glucose and its metabolic products in dormant tubers stored for a long enough time at a low positive temperature (cold sweetening) [17] clearly indicates that starch degradation enzymes are active during this period. This statement is corroborated by analysis of the expression pattern of potato genes encoding enzymes participating in starch utilization (Figure 1B; Table S1). Most of these genes retain their activities at every stage of the tuber life cycle including the dormancy stage. Under such conditions, potatoes are expected to adopt any strategy aiming to preserve as much starch as possible for the sprouting stage. One of the possible ways to resist tuber starch degradation is the persistent activity of enzymes for reverse reaction, namely starch biosynthesis. Our analysis of gene activities in potato tubers related to starch biosynthesis was consistent with this suggestion. Although the expression levels of most such genes were greatly reduced during dormancy stage, almost all genes nevertheless retained their activities to some detectable degree (Figure 1B; Table S1) (see also [20]).

However, it should be always taken into account that gene expression does not link directly to respective enzyme activity; additional regulation steps can exist at the levels of RNA transport and stability, as well as protein synthesis, modification, and stability. Therefore, it is very desirable to control the activity of the enzymes of interest irrespective of their cognate genes. One such enzyme playing a central role in starch formation is ADP-glucose pyrophosphorylase (AGPase) [2,21,22]. This enzyme catalyzes the rate-limiting reaction of starch biosynthesis, that is, the reversible conversion of glucose-1-phosphate to ADP-glucose, a starch precursor. The *AGPase* gene was found among potato genes expressed during tuber cold sweetening (Figure 1; Table S1) [14].

**Figure 2:**
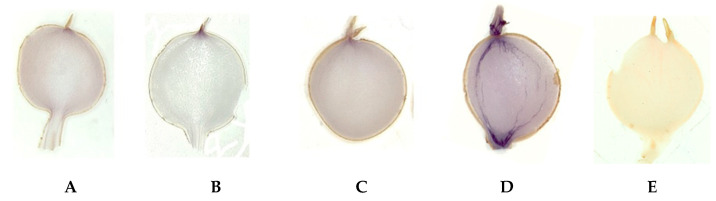
Histochemical staining of AGPase activity in potato microtubers. From left to right, sections of microtubers: (**A**) at the beginning of dormancy (0-week storage); (**B**–**D**) dormant microtubers stored for ~9 (**B**), 16 (**C**), or 25 (**D**) weeks (the latter at the end of dormancy period/starting sprouting). On the right, control for staining specificity (**E**) (without substrate ADP-glucose) shows plain background [22].

Using this enzyme as an example, we have checked whether dormant potato tubers retain starch-forming potential. Histochemical staining according to [22] confirmed that AGPase activity, though greatly reduced, persisted throughout the entire dormancy period, seemingly in the majority of tuber cells (Figure 2).

Thus, in dormant tubers stored in the dark at low (2–4 °C) temperature, starch biosynthesis occurs, or at least has an important prerequisite to occur, in parallel with limited but constant starch degradation. Such dynamic equilibrium between synthesis and breakdown was termed ‘starch cycling’ [23]. Initially, this cycling was attributed mainly to the starches in actively growing tissues. However, afterward, data accumulation led to the suggestion that starch cycling may not stop in tubers during the dormancy period either [15].

Therefore, in this connection a question arose: What happens to starch during prolonged storage of dormant tubers? Does this presumable ‘starch cycling’ lead to any kind of starch structure remodeling, or does it simply preserve the original structure without any significant changes?

To address this question, we conducted a comprehensive study of the structure and physicochemical properties of starches extracted from dormant tubers after long-term rest under typical storing conditions (2–4 °C, darkness, and limited humidity).

### 2.2. Starch Granule Morphology

**Figure 3:**
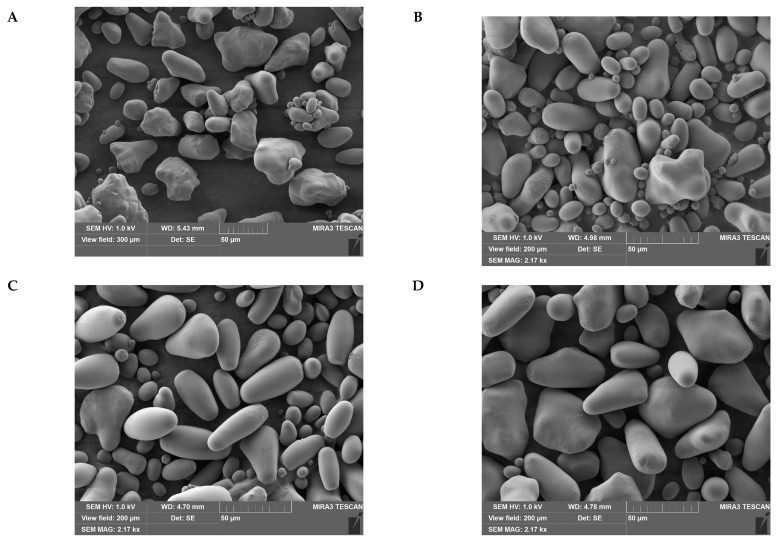
Scanning electron micrographs of potato starches extracted from tubers of WT (**A**,**B**) and *Arabidopsis phytochrome B* (*AtPHYB*)-transgenic (Dara 5) (**C**,**D**) potatoes, cultivated in vivo (bar = 50 µm). The time periods (in weeks) of tuber dormancy were following: 8 (**A**), 12 (**B**), 4 (**C**), and 8 (**D**).

Starch granule morphology was analyzed using scanning electron microscopy (SEM). As expected, all studied starches were markedly heterogeneous in granule size (Figure 3). The largest part of the granules was characterized by an oval shape with clear edges. However, among others, granules of irregular or cubic shape were also present. Similar granule variability was reported earlier for starches from potato and rice [24,25].

After prolonged dormancy of tubers, the visual proportion of large-sized granules increased for starches extracted from both control and *AtPHYB*-transgenic tubers, concomitantly with the proportion of granules of irregular shape. This finding was in agreement with [17] who reported average starch grain size increased during tuber storage due to reduction in the number of smaller starch grains.

These facts raise an interesting possibility that during dormancy of potato tubers, fusion of their starch granules occurs, mainly involving smaller granules. Such fusion presumably leads to average granule size increase, accompanied by raising the proportion of starch granules of irregular shape. It should be noted that the proportion of the latter was higher in starches from *AtPHYB*-transgenic than from non-transgenic tubers (Figure 3).

**Figure 4:**
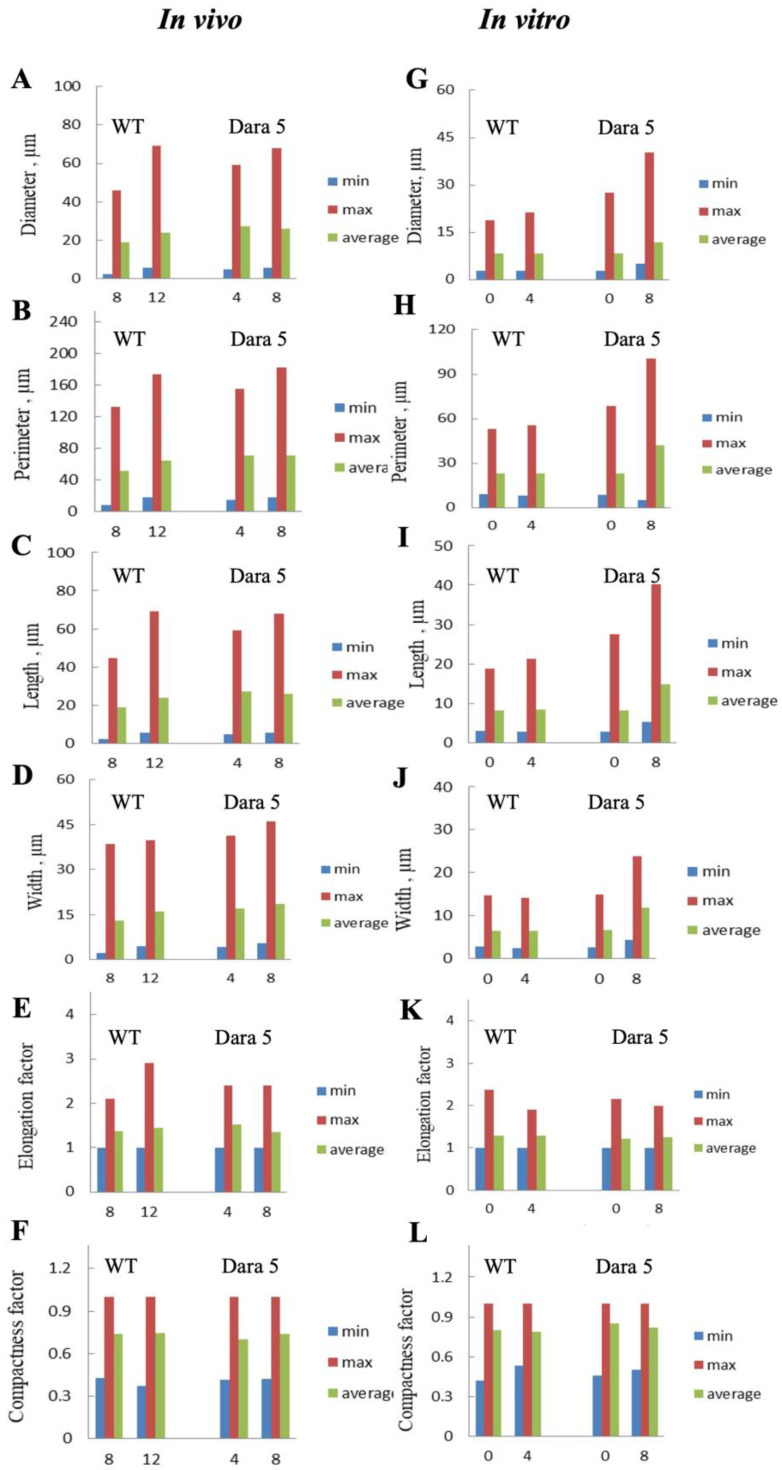
Granulometric parameters (diameter (**A**,**G**), perimeter (**B**,**H**), length (**C**,**I**), width (**D**,**J**), as well as elongation (**E**,**K**) and compactness (**F**,**L**) factors of starch granules from mature tubers of WT (non-transgenic) and *AtPHYB*-transgenic (Dara 5) potatoes, grown in vivo (**A**–**F**) or in vitro (**G**–**L**). X-axis of all plates indicates time of dormancy in weeks. For parameter description, see Section 3.

To accomplish a semi-quantitative comparison of starch granules from different experimental series, SEM granulometric analysis was performed. Some of the results are displayed in Figure 4.

According to SEM data, prolonged tuber storage can lead to increase in average starch granule size. This trend was manifested both in non-transgenic tubers in vivo kept resting for 4 weeks (from weeks 8 to 12) (Figure 4A–D) as well as Dara 5 tubers in vitro stored dormant for 8 weeks (from weeks 0 to 8) (Figure 4G–J). In this respect, the parameters related to granule size (diameter, perimeter, length, and width) demonstrated concerted changes. At the same time, parameters characterizing elongation and compactness of granules remained mostly unchanged (Figure 4E,F,K,L).

### 2.3. Starch Polymorphic Structure

It is well known that starches can adopt distinct polymorphic crystalline structures. In cereal plants, such as wheat and maize, amylopectins are characterized by monoclinic double chain packing, corresponding to A-type polymorphic structure. At the same time, starches from tuber crops, such as potato and yam (*Dioscorea alata*), are mainly characterized by hexagonal packaging of amylopectin double chains, corresponding to B-type polymorphic structure [26,27,28]. Starches of some species, for instance, from pea, adopt so-called C-type polymorphic structure, representing a mixture of A- and B-type structures [29,30].

The type of polymorphic structure seems to be a rather stable trait of starch from a defined species. In our previous study, ectopic expression of the *AtPHYB* gene in potato affecting some starch properties did not change the B-type polymorphic structure [19]. In the present study, we used wide-angle X-ray diffraction to assess the types of polymorphic structure of starches from dormant tubers stored for several weeks at 2–4 °C.

**Figure 5:**
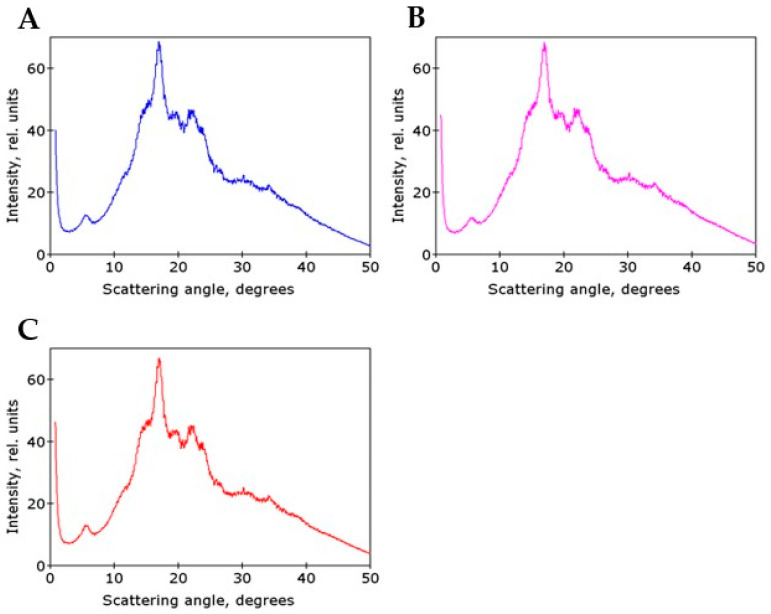
X-ray diffraction patterns of potato starches extracted from WT tubers cultivated in vivo without tuber storage (**A**) and after tuber storage at 2–4 °C during 8 (**B**) or 12 (**C**) weeks.

Three starch samples from non-transgenic potato tubers cultivated in vivo extracted from tubers without storage and from tubers kept dormant for 8 or 12 weeks were analyzed. X-ray diffraction patterns of these starch samples were very similar (Figure 5A–C). For all these samples, diffraction peaks were observed at the same position of 2θ scale, regardless of dormancy time. The position of these diffraction peaks corresponded to the starch polymorphic structure of B-type [31]. Investigated starch samples had almost the same degree of total crystallinity as was determined on the basis of their X-ray diffraction patterns. (For dormancy times 0, 8, and 12 weeks, total crystallinity was equal to 20.5%, 20.9%, and 20.6%, respectively.) Therefore, it may be concluded that during potato tuber dormancy the type of starch polymorphic structure and the total crystallinity do not change. This conclusion is also in accordance with a recent paper [32].

### 2.4. Starch Thermodynamic Properties

**Figure 6:**
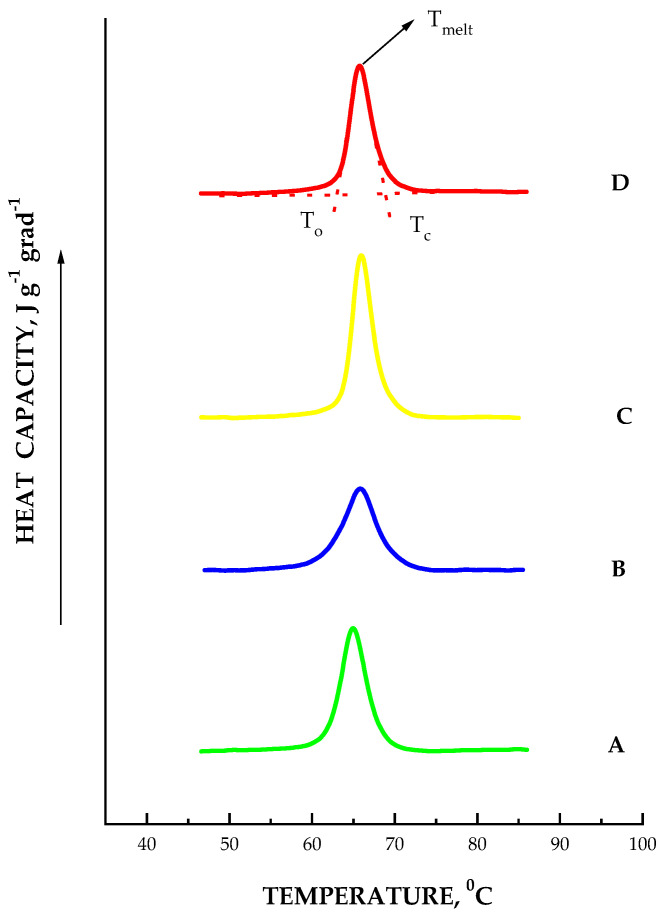
DSC thermograms of aqueous dispersions (0.3%, *w*/*w*) of potato starches extracted from WT (**A**,**B**) and *AtPHYB*-transgenic (Dara 5) (**C**,**D**) tubers grown in vivo and stored in dormancy state for various periods of time: (**A**–**D**)–8, 12, 4, and 8 weeks, respectively.

The thermogram curves of extracted starches are exemplified in Figure 6. It should be noted that the profile of thermograms for all the studied samples was typical for native potato starches [19,33,34,35,36]; therefore, not all thermograms are demonstrated. The endothermic peak on DSC thermograms is characterized by temperature transition (T_melt_), which is associated with endothermic, cooperative, irreversible thermodynamic transition known as gelatinization [37]. Such thermograms mainly reflect the melting of crystalline amylopectin lamellae (Figure 6).

Melting temperature can be considered as a measure of the starch structure stability [36,38]. The onset temperature and conclusion temperature of the transition were measured, respectively, as the corresponding intersections (T_0_, Tc) (see Figure 6, trace D). As follows from the data presented in Table 1, the melting thermodynamic parameters (T_melt_ and H_melt_) of investigated starches decreased with increasing of the dormant period. The established fact of the melting temperature decrease for starches during the dormant period is correlated with data in the literature [39]. At the same time, the width (ΔT = T_0_ − Tc) of the starch transition increases with an increase in the dormancy period of tubers cultivated both in vivo and in vitro, which indicates the formation of less perfect crystalline structures, which reduces starch crystallinity [38,40,41]. In our study, the starch crystalline structures during tuber dormancy became less stable and less homogenous compared to the structures of the corresponding starches from tubers that were at rest for much less time. It should be noted that the greatest changes in the width ΔT were observed in starches from *AtPHYB*-transgenic (Dara 5) potato tubers cultivated in vitro with dormancy over a period of 8 weeks. In general, the width of the melting transition of potato starches increased during storage of both in vivo and in vitro cultured tubers, indicating the formation of less perfect crystalline structures in the starch. It is possible that the amylopectin double helices in starch from potato tubers elongate during storage without increasing their number, which may explain the absence of changes in X-ray diffraction patterns [42].

From the above graphics it can be seen that the melting curves for all the studied aqueous dispersions were, on the whole, symmetrical with respect to the maximum melting point. However, with an increase in the resting time of tubers, the melting curves became less symmetrical. This indicates certain structural changes in the starch, which became more pronounced as the resting time of the tubers was extended. Calculation of thermodynamic parameters according to a one-stage model showed (Table 1) that van’t Hoff enthalpy also decreased with an increase in the resting time of tubers for all starch samples studied.

As a rule, thickness of the crystalline lamellae of starches is calculated from SAXS data (for example [43,44]). The data on the thickness of the crystalline lamella obtained from SAXS data are determined based on model calculations, and the values of the thickness of the lamella may depend on the choice of the model used. It was shown early that the value of the crystalline lamella thickness of starches obtained from SAXS and DSC data were in good agreement (for example, [45,46]). Taking this fact into account, we determined the thickness of crystalline lamella from DSC data.

The trend to decrease the value of the thickness of the crystalline lamella of starches from WT tubers cultivated both in vivo and in vitro with an increase in the resting time of the tubers was observed. In the case of starches extracted from tubers of *AtPHYB* plants, the thickness of the crystalline lamellae, on the contrary, increased with the length of time the tubers were at rest (Table 1). The reasons for the multidirectional changes in the thickness of the crystalline lamellae of starches from WT and transgenic plants are not yet clear, but the very fact of a change in this starch parameter in resting tubers is important here.

It was shown that the melting point of semi-crystalline synthetic polymers can be calculated using the Thomson–Gibbs equation [40,47]:

T_melt_ = T^0^_melt_ (1 − 2γ_i_/(ΔH^0^_melt_ ρ_crl_L_crl_)) (1)

where T^0^_melt_ and ΔH^0^_melt_ are the melting temperature and the melting enthalpy, respectively, of a hypothetical crystal with unlimited size (a perfect crystal) or such crystals for which the role of free surface energy in comparison with volume energy can be neglected, respectively; γ_i_ is the relevant surface free energy; ΔH^0^_melt_ is the experimental enthalpy; and ρ_crl_ and L_crl_ are the density and the thickness of the crystalline lamellae, respectively.

Based on Equation (1), one can obtain with Equations (2) and (3) useful information regarding the thermodynamic parameters of crystalline lamellae melting, namely, surface entropy *q_i_* and enthalpy *s_i_* of lamellae end faces [40,47]:

*q_i_* = (ΔH^0^_melt_ − ΔH_melt )_ L_crl_/2.5 (2)

*s_i_* = (*q_i_* − γ_i_)/T_melt_ (3)

It is well known that the melting of a polymer crystal begins with the melting of defects in an amorphous lamella, and, therefore, the surface entropy value of the end faces of the crystalline lamellae is proportional to the number of defects in starch granule crystalline structures [40,48]. It is possible to qualitatively assess defects using the differential scanning microcalorimetry method. It should be noted that in the case of starches, such defects can result from amylose transition chains, representing single twisted helices having an unordered conformation, and molecularly ordered structures, consisting of double helices and located between crystallites in an amorphous lamellae, as well as amylopectin B chains, also located in amorphous lamellae [47,49].

The accumulation of such defects is accompanied by the formation of crystallites with a more ‘mellow’ surface and a decrease in the melting point. The thermodynamic parameters of the surface of the end faces of the crystalline lamellae of the studied starches were evaluated (Table 2). For calculations, the T^0^_melt_ and ΔH^0^_melt_ values were approximated with those relevant to B-type spherulitic crystals, namely, T^0^_melt_ = 346.8 K and ΔH^0^_melt_ = 35.5 J g^−1^, and the value of density (ρ_crl_) of B-type structures 1.40 g cm^−3^ [50]. The values of L_crl_, ΔH_melt_, and T_melt_ were directly evaluated from the experimental DSC traces obtained for the investigated starches (Table 2).

From the above data, it is seen (Table 2) that the value of the surface entropy of the end faces of crystalline lamellae increases for starches extracted from tubers cultivated in vivo and in vitro and having a quite long period at rest. This is accompanied by a decrease in the melting temperature (Table 1). This fact allows us to conclude that when tubers are at rest, defects accumulate in the starch structure, which is consistent with data from the literature [17]. Given that the value of the surface entropy of the end faces of crystalline lamellae of starches from *AtPHYB*-transgenic potato tubers is higher than the corresponding values for starches from untransformed tubers, we can assume that starches from *AtPHYB*-transgenic tubers accumulate more defects during the dormancy period than starches from non-transgenic tubers.

Given that the DSC thermograms of the studied starches are characterized by some asymmetry of melting peaks, we can assume that this is a manifestation of the presence of more than one independent transition of crystalline structures with different melting points. The results of deconvolution of DSC thermograms of starches from untransformed and *AtPHYB*-transgenic tubers are shown in Table 3. The deconvolution of the heat capacity curves was carried out in accordance with the model of two independent ‘all-or-nothing’ transitions. Figure 5 shows examples of the results of deconvolution of DSC thermograms for starches extracted from transgenic tubers of the Dara 5 variant cultivated in vitro and in vivo and that have been at rest for various periods of time.

**Figure 7:**
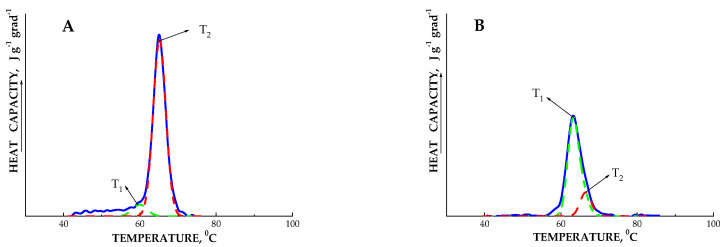

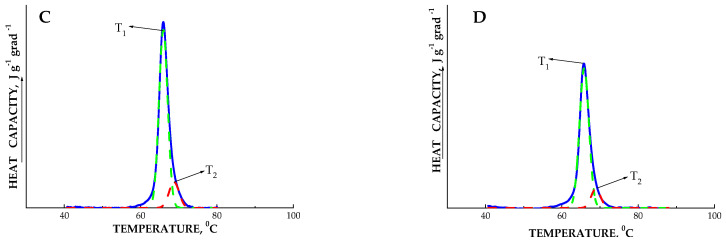
Experimental DSC thermograms (solid line) and deconvolution results (dashed line) for potato starches extracted from *AtPHYB*-transgenic Dara 5 tubers cultivated in vitro (**A**,**B**) or in vivo (**C**,**D**) and then stored for various periods of time: 0 (**A**), 8 (**B**), 4 (**C**), or 8 (**D**) weeks. T_1_ and T_2_ are the melting points of low- and high-temperature structures, respectively (see Table 3).

From the data in Table 3 and Figure 7, it follows that upon melting of all the studied starches, two independent transitions occurred, which were caused by structures with different melting points. As noted above, the melting of crystalline lamellae begins with the destruction of defective sites in starch crystals, such as chain segments and amylopectin loops [51]. The low-temperature transition obviously reflects melting of crystalline lamellas of a more defective structure compared to structures that are characterized by a high-temperature transition. From the presented results, it follows that during dormancy, the proportion of low-temperature, unordered structures increases; moreover, the proportion of these defects in starches from *AtPHYB*-transformed tubers is greater than in the untransformed variant (Table 3).

**Figure 8:**
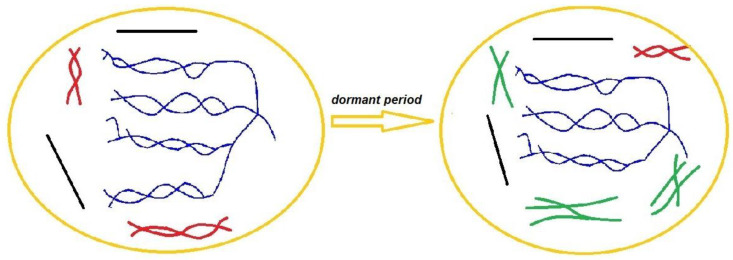
Schematic illustration of starch granule remodeling during storage of potato tubers where amylose chains (black lines), amylopectins (blue lines), molecular ordered structures (red lines), and unordered chains (green lines) are shown.

Thus, the data obtained from DSC thermograms and their analysis show that the storage process of potato tubers is accompanied by an alteration of the crystalline structure of starches from these tubers and the accumulation of additional defective structures in them, which is shown schematically in Figure 8.

### 2.5. General Considerations

It is likely that ectopic expression of the *PHYB* gene from *Arabidopsis thaliana* increases the occurrence of defects in comparison with starch from non-transgenic tubers [19,33], which leads to differences in the thermodynamic parameters of starch melting from tubers upon their prolonged rest. This effect is observed in plants grown both in vitro and in vivo. In this case, the melting points of the low-temperature transition of crystalline lamellae decreased with an increase in the duration of tuber dormancy. Apparently, in the starches extracted from tubers (especially *AtPHYB*-transgenic), which were kept at rest, some irregular (unordered) structures accumulated.

Data were reported that evidence that irregular amorphous sites are most suitable for enzymatic starch hydrolysis [52,53]. Potato and other B-type starches are digested by an exocorrosion process, starting from the granule surface. Granules that have perfectly smooth surfaces are less susceptible to digestion. As amylases are able to penetrate the surface through the pores, one would expect preferential hydrolysis of amorphous regions prior to ordered crystallites. The favored digestion of amorphous regions seems to be reasonable since double helices of crystalline lamellae cannot be digested unless they are untwisted [53]. Indeed, according to one study, in the course of starch enzymatic treatment, α-amylase preferentially attacked the amorphous region of the granule (amylose), while the crystalline structure was minorly affected. Accordingly, water firstly entered the amorphous region before penetrating the crystalline region [52]. In addition, the changes we detected in size and shape of starch granules also may not be indifferent to starch hydrolysis rate. In fact, granule size and shape are known to influence starch digestibility and glucose outcome, mostly because the shift from polyhedral to spherical shape affects the specific surface area. In a study of starch enzymatic hydrolysis, it was found that smaller starch granules yielded relatively less glucose than larger granules [54]. According to recent literature, the resting period affects the starch structure in dormant tubers not only of potato (B-type polymorphic structure), but also of Chinese yam *Dioscorea opposita* Thunb (A-type polymorphic structure) [55].

The amorphous, unordered structures of potato starch melting at low temperature can serve as initial sites of active starch hydrolysis needed for tuber sprouting. This sprouting should proceed during a favorable season, which may last not too long. Therefore, the more primary sites of starch hydrolysis exist in a tuber, the more material and energy are provided for the concerted growth of sprouts. Hence, the starch structure remodeling during the dormancy period described here can have a clear physiological meaning: a preadaptation to the upcoming physiological stage—tuber sprouting—of the tuber life cycle. In other words, this means that during storage, dormant potato tubers gradually transform the starch structure in order to provide the hydrolysis surge required at the upcoming sprouting stage.

Our pioneering finding that the *PHYB* gene constitutively expressed in potato may affect starch structure in tubers [19] seemed rather enigmatic at the time. However, this finding was corroborated soon thereafter by the study of photoreceptor mutants (including *phyA* and *phyB* plants) of *Arabidopsis* showing a metabolic link between photoreceptors and starches [56]. Plants defective in phytochromes A and/or B accumulated less starch than their non-mutant counterparts. Of course, unlike potato, *Arabidopsis* cannot produce tubers, so the reasonable question arises, by what mechanism may *PHYB* affect metabolic processes in the tuber, in particular the process of starch formation. This interesting task remains to be addressed. As evidence in favor of such a relationship, we can cite the data from Schittenhelm et al. (2004) [57] who showed that in Dara 5 plants in vivo, the relative content of nitrogen (N) in tubers was shifted significantly compared to untransformed potatoes.

## 3. Materials and Methods

### 3.1. Experimental Plants

The study was carried out with WT (control) potatoes (*Solanum tuberosum* L.) cv. Dėsirėe, as well as with two *AtPHYB*-transgenic lines derived from these varieties: Dara 5 and Dara 12 [58]. These transgenic lines expressing the *A. thaliana phytochrome B* gene (*AtPHYB*), under the control of the constitutive 35S CaMV promoter, were kindly provided by Prof. C. Gatz (Goettingen University, Germany). The authors of the transformants showed that the Dara 12 line expresses the *AtPHYB* transgene more strongly than the Dara 5 line.

Plants were propagated with single-node cuttings and grown in vitro in sterile tubes on agarized MS medium under long-day conditions at a temperature of 22–24 °C. Tuberization was induced by high (5%) sucrose content in the medium. A part of the obtained microtubers (in vitro tubers) was planted in the pots with soil and put under long-day conditions at a temperature of 22–24 °C, giving rise to full-grown plants that formed tubers (in vivo tubers). For this study, dormant tubers and microtubers of the same year were stored in the dark at a temperature of 2–4 °C for various periods (0–25 weeks). Starch was extracted from tubers and microtubers according to the standard procedure [59] and was investigated using the physicochemical methods listed below.

### 3.2. In Situ Staining of ADP-Glucose Pyrophosphorylase (AGPase) Activity

Histochemical staining was based on the coupling of oxidation NADH to the reduction of nitroblue tetrazolium (NBT), which resulted in the precipitation of blue tetrazolium salt. In situ staining of AGPase activity was performed as described [22] with some modifications. In brief, non-transgenic tubers of different physiological states (developing, dormant, and sprouting) were used, with 5 tubers for each assay. Sections of 140 µm thickness were cut with a sledge microtome. The sections were immediately placed in the fixation mixture (2% paraformaldehyde, 2% soluble polyvinylpyrrolidone 40, and 0.01 M DTT) and kept at 4 °C for 1 h. After fixation, sections were rinsed in water at least five times with 1 h intervals, one time overnight, at 4 °C. AGPase activity was visualized by incubating the tuber sections in 1 mL of reaction mixture at 30 °C for 30 min. The reaction mixtures used for AGPase activity testing and respective controls (the same mixture but without specific substrate) were described in detail in [22]. After the incubation period, the sections were transferred to distilled water to stop the reaction and kept at 4 °C until further investigation.

### 3.3. Scanning Electron Microscopy (SEM)

Morphological changes of the starch granule were investigated using scanning electron microscopy (SEM). Photos of starch granules were obtained with a scanning electron microscope Mira3 LMU (Tescan, Brno, Czech Republic) at room temperature in the conditions of high vacuum with accelerating voltage 500 V.

Size and morphology of the potato starch granules were measured or estimated using an extended suite of morphometric algorithms under the ‘Altami Studio’ (version 3.4.0) licensed software GUI. Besides the standard granulometric characteristics, a number of dimensionless shape parameters were analyzed and compared. These parameters included elongation factor, calculated as the ratio of equivalent length and width, as well as compactness factor, calculated as the ratio of the area of a figure to the area of its circumscribing circle. Each value represented an average of 100–200 granule measurements.

### 3.4. X-ray Diffraction

The type of polymorphic crystalline structure of starches was characterized using the method of X-ray diffraction. The starches were studied in an air-dried state at ambient room temperature. XRD patterns were taken in transmission mode using the X-ray diffractometer of a local design described elsewhere (Cu Kα radiation) [60]. Registration of diffraction patterns was performed with a linear position-sensitive detector installed with an inclination toward the sample at 20°. The sample-to-detector distance was 92 mm. The width of the primary X-ray beam in the sample plane and the width of the detector window were restricted to 4 mm. X-ray diffraction intensity was corrected for background scattering measured with an empty sample holder and normalized to a starch sample mass.

A starch degree of crystallinity *C* was calculated as in [42] with the equation:

(4) $$C=\frac{\int_{2\theta_{1}}^{2\theta_{2}}\left[I\left(2\theta \right)-I_{b}\left(2\theta \right)-I_{a}\left(2\theta \right)\right]d\left(2\theta \right)}{\int_{2\theta_{1}}^{2\theta_{2}}\left[I\left(2\theta \right)-I_{b}\left(2\theta \right)\right]d\left(2\theta \right)}\cdot 100\%$$

carrying out intensity integration in the range of scattering angles 2*θ* from 3° to 50°. In this equation, *I*(2*θ*) is experimental XRD intensity of a starch sample, *I_b_*(2*θ*) is a baseline intensity assumed to be a straight line passing through experimental intensity points at 2*θ* = 3° and 2*θ* = 50°, and *I_a_*(2*θ*) is the intensity of the starch amorphous part determined on the basis of the experimental XRD pattern of amorphous starch.

### 3.5. High-Sensitivity Differential Scanning Microcalorimetry

The melting thermodynamic parameters of 0.3% (DW) starch aqueous dispersions were determined using a high sensitivity differential scanning microcalorimeter DASM-4 (NPO “Biopribor”, Pushchino, Moscow region, Russia) in the 20–100 °C temperature range with a 2 K min^−1^ heating rate and 2.5 bar excess pressure. The volume of the investigated sample was 0.5 cm^3^ in sealed cells. Deionized water (Millipore Direct-Q3) with a resistivity of 18.2 μS·cm^−1^ at a temperature of 25 °C was used as a reference solution. The heat capacity was calibrated using the Joule–Lenz effect for each run. Corrections for dynamic lag and residence of the samples in calorimetric cell were not obligatory under the conditions used [61,62].

The average values of the thermodynamic parameters were determined as described elsewhere [61,63] using three measurements at 95% significance level and converted to values per mole of anhydroglucose units (162 g mol^−1^).

The melting process of starches can be considered in the first approximation as quasi-equilibrium [42,61,63,64,65]. Therefore, this fact allows applying a one-stage melting model, in which the melting process of starches is considered as an equilibrium reaction between native and molten states. This model was also applied for evaluation of the cooperative parameter and thickness of crystalline lamellae [61,63,65].

Values of van’t Hoff enthalpy (ΔH^vH^) were calculated according to [64,65], using the following equation:

ΔH^νH^= 2 R^1/2^ T_melt_(C_p_^*^ − 0.5ΔC_p_^exp^) ^½^ (5)

where R is the gas constant; T_melt_ is melting temperature of the crystalline lamellae, K; C_p_^*^ is the maximum ordinate of the DSC trace; and ΔC_p_^exp^ is the difference in the heat capacities of phase native (crystalline) and molten (amorphous) state.

For starches with a symmetric DSC signal, a previously reported method [35,49,64] was used to evaluate the melting cooperative unit, ν:

ν = (ΔH^vH^)/(ΔH_melt_) (6)

where ΔH_melt_ is the experimental melting enthalpy of the crystalline lamellae.

The thicknesses of the crystalline lamellae (L_crl_) were calculated according to Equation (7) [64]:

L_crl_ = 0.35ν (7)

where 0.35 nm corresponds to a pitch height per anhydroglucose residue in the double helix of amylopectin [27,66]. 

In the cases of starches possessing asymmetric melting endotherms, the Peak Fit program (AISN Software Incorporated, version 4) was applied to deconvolute the overall melting endotherms and calculate the thermodynamic parameters corresponding to the components separately [63].

### 3.6. Analysis of Starch-Related Gene Expression in Potato

In our work, we identified most important genes of the currently proven components of potato starch biosynthesis and degradation in agreement with [67,68,69]. Data from the NCBI (GeneBank) database were used as the basis. The genes/proteins of heterozygous diploid RH89-039-16 (RH) were searched with the BLASTPsuit service, using sequences of corresponding Arabidopsis proteins. The data in Table S1 are given for SolTub_3.0 version (GCF_000226075.1). The data obtained were compared with the data available in Phytozome 13 for *Solanum tuberosum* v.6.1. Information on the localization of genes on potato chromosomes was obtained from there. This database was also used to retrieve information about gene expression in various organs (http://spuddb.uga.edu/dm_v6_1_download.shtml (accessed on 6 April 2023). Only genes for which there was reason to assume participation in starch biosynthesis and degradation were considered.

### 3.7. Statistical Analysis

All essential experiments were repeated at least twice. In the cases where it was possible, statistics were employed (see Table 1 and Table 2). Data given represent mean values with standard errors (SE). *p* < 0.05 was considered according to Student’s criterion as statistically significant).

## 4. Conclusions

It was previously believed that in dormant tubers stored at low-positive temperatures (2–4 °C) starch is mostly inert and that only a very small part of the starch is broken down to mobile carbohydrates (cold sweetening) to satisfy minimal metabolic needs of tuber cells (negligible respiration and glycolysis). Later on, it became clear that starch in dormant tubers not only degrades, but also can be synthesized by using its decay products during a process termed the ‘starch cycle’. In our study, we confirmed the possibility of starch cycling in dormant tubers. In addition, by means of sensitive physicochemical methods we found evidence for remodeling of the starch structure, which likely affects both the structure of amylopectin (the thickness of the crystalline lamellae) as well as the amylose-to-amylopectin ratio (more amorphous components).

The argument attesting to the fact that these changes originated in dormant tubers during the storage period was the positive dependence of structural defect accumulation on storage duration, which was observed for tubers grown both in vivo (plants in the soil) and in vitro (axenic plants in tubes). In addition to changes in the starch’s fine structure, global changes in the shape and size of the starch granules during tuber dormancy were uncovered. Such changes, which can be explained by the fusion of, predominantly, small granules, point to the lability of the structure of starches in this period both at the micro and macro levels. Tuber storage at the dormancy stage affects micro and macro starch structures not only in potato (B-type polymorphic structure) but also in Chinese yam (A-type polymorphic structure) as well. However, despite of all these alterations, an important parameter of the starch structure was kept unchanged: that was a species-specific polymorphic structure. Even in *AtPHYB* transformants constitutively expressing the *Arabidopsis phytochrome B* gene, the starch retained its original polymorphic structure, despite the fact that starch of such tubers usually accumulates a higher proportion of unordered components. The problem of metabolic relationships between ectopically expressed phytochrome and starch structure in tuber cells represents a new prospect for further studies. Taken together, our data led us to the conclusion that during the whole dormancy period of potato tubers, their starch remains metabolically and structurally labile. It is assumed that the accumulated amorphous starch components and alterations in starch granule size and shape have physiological significance as prerequisites for massive starch hydrolysis, which will be required at the upcoming tuber sprouting stage.

## Figures and Tables

**Table 1:** Thermodynamic parameters: melting temperature (T_melt_), the temperature range of gelatinization (ΔT), melting enthalpy (ΔH_melt_), van’t Hoff enthalpy (ΔH^νH^), cooperative melting units (ν), and thickness of crystalline lamellae (L_crl_) of potato starches from untransformed (WT) and *Arabidopsis phytochrome B(AtPHYB*)-transgenic (Dara 5, Dara 12) tubers grown in vitro or in vivo and stored dormant for various periods of time *.

Variant	Cultivation Conditions	Dor- mancy Time, Weeks	T_melt_, °C	Δ T	ΔH_melt_, kJ/mol	ΔH^vHof^, kJ/mol	ν, Anhydro- Glucose Units	L_crl_, nm
WT	in vitro	0	64.4 ± 0.0	7.7	2.9 ± 0.1	40.5 ± 0.1	14.0 ± 0.1	4.9 ± 0.1
		4	64.4 ± 0.0	8.0	2.9 ± 0.1	39.9 ± 0.1	13.8 ± 0.1	4.8 ± 0.1
		16	64.0 ± 0.0	8.8	2.5 ± 0.1	30.6 ± 0.1	12.5 ± 0.1	4.3 ± 0.1
	in vivo	8	65.0 ± 0.1	7.6	3.5 ± 0.1	55.3 ± 0.2	15.8 ± 0.1	5.5 ± 0.1
		12	65.8 ± 0.1	9.1	3.0± 0.4	43.8 ± 1.1	15.1 ± 1.5	5.3 ± 0.5
Dara 5	in vitro	0	65.1 ± 0.1	7.9	2.7 ± 0.1	45.5 ± 0.3	17.6 ± 0.3	6.1 ± 0.1
		8	63.4 ±0.0	9.2	1.7 ± 0.1	33.8 ± 0.2	20.2 ± 0.1	7.1 ± 0.1
	in vivo	4	66.0 ± 0.1	5.0	3.8 ± 0.2	65.1 ± 1.6	16.5 ± 0.2	5.8 ± 0.1
		8	65.7 ± 0.1	6.0	2.6 ± 0.1	52.6 ± 0.6	20.2 ± 0.1	7.0 ± 0.1
Dara 12	in vitro	0	67.0 ± 0.1	7.3	2.8 ± 0.1	44.9 ± 0.3	16.0 ± 0.7	5.6 ± 0.3
		7	66.1 ± 0.1	7.6	1.7 ± 0.1	38.7 ± 0.1	22.8 ± 0.1	7.9 ± 0.2
		10	65.4 ± 0.1	8.2	1.7 ± 0.1	34.7 ± 0.1	20.3 ± 0.1	7.9 ± 0.2

* Deviations less than 0.05 were rounded off to 0.0. ΔT = T_c_ − T_0_, where T_0_–onset temperature, T_c_ conclusion temperature of gelatinization.

**Table 2:** Thermodynamic parameters: free surface energy (*γ_i_*), enthalpy (*q_i_*), and entropy (*s_i_*) of the melting of the end faces of crystalline lamellae of starches from untransformed (WT) and *AtPHYB*-transgenic (Dara 5 and Dara 12) tubers grown in vitro or in vivo and stored dormant for various periods of time.

Variant	Cultivation Conditions	Dormancy Time, Weeks	*γ_i_* × 10^7^ J cm^−2^	*q_i_* × 10^7^ J cm^−2^	*s_i_* × 10^7^ J cm^−2^ grad^−1^
WT	in vitro in vivo	0	3.233 ± 0.067	47.27 ± 0.67	0.131 ± 0.002
		4	3.301 ± 0.067	48.26 ± 0.71	0.133 ± 0.002
		16	3.020 ± 0.070	48.29 ± 0.36	0.134 ± 0.001
		8	3.468 ± 0.002	42.80 ± 0.19	0.116 ± 0.005
		12	3.042 ± 0.325	52.23 ± 12.55	0.145 ± 0.036
Dara 5	in vitro	0	3.856 ± 0.053	66.26 ± 1.92	0.185 ± 0.006
		8	5.342 ± 0.075	99.39 ± 1.05	0.280 ± 0.003
	in vivo	4	3.348 ± 0.154	40.67 ± 7.23	0.110 ± 0.021
		8	4.066 ± 0.004	75.32 ± 0.93	0.210 ± 0.003
Dara 12	in vitro	0	2.656 ± 0.073	56.65 ± 3.41	0.160 ± 0.099
		7	4.357 ± 0.054	110.56 ± 0.07	0.313 ± 0.005
		10	4.272 ± 0.069	99.36 ± 0.35	0.281 ± 0.000

**Table 3:** Results of deconvolution of DSC thermograms of potato starches from untransformed (WT) and *AtPHYB*-transgenic (Dara 5, Dara 12) plants cultivated in vitro or in vivo. Tubers were kept in a dormancy state for various times. α1 and α2 are proportions of melting enthalpies of structural elements melting at low and high temperatures, respectively, from the total melting enthalpy.

Variant	Cultivation Conditions	Dormancy Duration, Weeks	Melting Temperature (°C) and Proportion (%)			
			Low Temperature Transition		High Temperature Transition	
			T_1_, °C	α_1_, %	T_2_, °C	α_2_, %
WT	in vitro	0	58.1	11.5	64.4	88.5
		4	61.3	14.0	64.7	86.0
		16	61.7	19.3	64.1	80.7
	in vivo	8	62.6	12.2	65.2	87.8
		12	62.3	15.3	66.1	84.7
Dara 5	in vitro	0	62.5	12.4	65.3	87.6
		8	63.2	83.4	66.5	16.6
	in vivo	4	66.0	85.5	68.8	14.4
		8	65.9	88.0	68.9	12.0
Dara 12	in vitro	0	61.5	9.1	66.9	90.9
		7	66.1	87.4	68.9	12.6
		12	65.8	90.4	68.7	9.6

## Data Availability

Not applicable.

## Supplementary Materials

The supporting information can be downloaded at: https://www.mdpi.com/article/10.3390/ijms24098397/s1.

## References

[1] MacNeill G.J., Mehrpouyan S., Minow M.A.A., Patterson J.A., Tetlow I.J., Emes M.J. (2017). Starch as a source, starch as a sink: The bifunctional role of starch in carbon allocation. J. Exp. Bot..

[2] Zeeman S.C., Kossmann J., Smith A.M. (2010). Starch: Its metabolism, evolution and modification in plants. Annu. Rev. Plant Biol..

[3] Aksenova N.P., Sergeeva L.I., Konstantinova T.N., Golyanovskaya S.A., Kolachevskaya O.O., Romanov G.A. (2013). Regulation of potato tuber dormancy and sprouting. Russ. J. Plant Physiol..

[4] Ozeretskovskaya O.V., Chailakhyan M.K., Mokronosov A.T. (1990). Cellular and molecular mechanisms of potato immunity. Regulation of Growth and Development of Potatoes.

[5] Sukhova L.S., Korableva N.P., Chailakhyan M.K., Mokronosov A.T. (1990). Regulation of potato tubers dormancy and their disease resistance by altering hormonal balance using the ethylene donors. Regulation of Growth and Development of Potatoes.

[6] Suttle J.C., Vreugdenhil D. (2007). Dormancy and sprouting. Potato Biology: Advances and Perspectives.

[7] Lang G.A., Early J.D., Martin G.C., Darnell R.L. (1987). Endo-, para-, and eco- dormancy: Physiological terminology and classification for dormancy research. Hort. Sci..

[8] Sheikh F.R., Jose-Santhi J., Kalia D., Singh K., Singh R.K. (2022). Sugars as the regulators of dormancy and sprouting in geophytes. Ind. Crop. Prod..

[9] Hemberg T., Li P.H. (1985). Potato rest. Potato Physiology.

[10] Aksenova N.P., Konstantinova T.N., Golyanovskaya S.A., Kossmann J., Willmitzer L., Romanov G.A. (2000). Transformed potato plants as a model for studying the hormonal and carbohydrate regulation of tuberization. Russ. J. Plant Physiol..

[11] Shan J., Song W., Zhou J., Wang X., Xie C., Gao X., Xie T., Liu J. (2013). Transcriptome analysis reveal novel genes potentially involved in photoperiodic tuberization in potato. Genomics.

[12] Xu X., Vreugdenhil D., van Lammeren A.A.M. (1998). Cell division and cell enlargement during potato tuber formation. J. Exp. Bot..

[13] Fernie A.R., Willmitzer L. (2001). Molecular and biochemical triggers of potato tuber development. Plant Physiol..

[14] Menéndez C.M., Ritter E., Schäfer-Pregl R., Walkemeier B., Kalde A., Salamini F., Gebhardt C. (2002). Cold sweetening in diploid potato: Mapping quantitative trait loci and candidate genes. Genetics.

[15] Sergeeva L.I., Claassens M.M.J., Jamar D.C.L., van der Plas L.H.W., Vreugdenhil D. (2012). Starch-related enzymes during potato tuber dormancy and sprouting. Russ. J. Plant Physiol..

[16] Solomos T., Mattoo A.K., Razdan M.K., Mattoo A.K. (2005). Starch-sugar metabolism in potato (*Solanum tuberosum* L.) tubers in response to temperature variations. Genetic Improvement of Solanaceous Crops. Volume I: Potato.

[17] Barichello V., Yada R.Y., Coffin R.H., Stanley D.W. (1990). Low temperature sweetening in susceptible and resistant potatoes: Starch structure and composition. J. Food Sci..

[18] Vaananen T., Ikonen T., Jokela K., Serimaa R., Pietila L., Pehu E. (2003). X-ray scattering study on potato (*Solanum tuberosum* L.) cultivars during winter storage. Carbohydr. Polym..

[19] Wasserman L.A., Sergeev A.I., Vasil’ev V.G., Plashchina I.G., Aksenova N.P., Konstantinova T.N., Golyanovskaya S.A., Sergeeva L.I., Romanov G.A. (2015). Thermodynamic and structural properties of tuber starches from transgenic potato plants grown in vitro and in vivo. Carbohydr. Polym..

[20] Kulakova A.V., Efremov G.I., Shchennikova A.V., Kochieva E.Z. (2022). Dependence of the content of starch and reducing sugars on the level of expression of the genes of β-amylases StBAM1 and StBAM9 and the amylase inhibitor StAI during long-term low-temperature storage of potato tubers. Vavilov J. Genet. Breed..

[21] Lloyd J.R., Kossmann J. (2019). Starch trek: The search for yield. Front. Plant Sci..

[22] Sergeeva L.I., Vreugdenhil D. (2002). In situ staining of activities of enzymes involved in carbohydrate metabolism in plant tissues. J. Exp. Bot..

[23] Appeldoorn N.J.G., de Bruijn S.M., Koot-Gronsveld E.A.M., Visser R.G.F., Vreugdenhil D., van der Plas L.H.W. (1999). Developmental changes in enzymes involved in the conversion of hexose-phosphate and its subsequent metabolites during early tuberisation of potato. Plant Cell Environ..

[24] Jaspreet S., Narpinder S. (2003). Studies on the morphological and rheological properties of granular cold water soluble corn and potato starches. Food Hydrocoll..

[25] Zhou X., Ying Y., Hu B., Pang Y., Bao J. (2018). Physicochemical properties and digestibility of endosperm starches in four indica rice mutants. Carbohydr. Polym..

[26] Yu Y., Han F., Huang F., Xiao L., Cao S., Liu Z., Thakur K., Han L. (2022). Physicochemical properties and molecular structure of starches from potato cultivars of different tuber colors. Starch/Stärke.

[27] Buléon A., Colonna P., Planchot V., Ball S. (1998). Starch granules: Structure and biosynthesis. Inter. J. Biol. Macromol..

[28] Jiang S., Cen J., Zhou Y., Wang Y., Wu D., Wang Z., Sun J., Shu X. (2023). Physicochemical characterizations of five *Dioscorea alata* L. starches from China. Int. J. Biol. Macromol..

[29] Bogracheva T.Y., Morris V.J., Ring S.G., Hedley C.L. (1998). The granular structure of C-type pea starch and its role in gelatinization. Biopolymers.

[30] Bertoft E. (2017). Understanding starch structure: Recent progress. Agronomy.

[31] Cairs P., Bogracheva T., Ring S.G., Hedley L.L., Morris V.J. (1997). Determination of the polymorphic composition of smooth pea starch. Carbohydr. Polym..

[32] Ye F., Xiao L., Liang Y., Zhou Y., Zhao G. (2019). Spontaneous fermentation tunes the physicochemical properties of sweet potato starch by modifying the structure of starch molecules. Carbohydr. Polym..

[33] Aksenova N.P., Wasserman L.A., Sergeeva L.I., Konstantinova T.N., Golyanovskaya S.A., Krivandin A.V., Plashchina I.G., Blaszczak W., Fornal J., Romanov G.A. (2010). Agrobacterial *rol* genes modify thermodynamic and structural properties of starch in microtubers of transgenic potato. Russ. J. Plant Physiol..

[34] Donovan J. (1978). Phase transition of the starch water system. Biopolymers.

[35] Protserov V.A., Karpov V.G., Kozhevnikov G.O., Wasserman L.A., Yuryev V.P. (2000). Changes of thermodynamic and structural properties of potato starches (Udacha and Acrosil varieties) during biosynthesis. Starch/Stärke.

[36] Jagadeesan S., Govindaraju I., Mazumder N. (2020). An insight into the ultrastructural and physiochemical characterization of potato starch: A review. Am. J. Potato Res..

[37] Chakraborty I., Govindaraju I., Kunnel S., Managuli V., Mazumder N. (2023). Effect of storage time and temperature on digestibility, thermal, and rheological properties of retrograded rice. Gels.

[38] Vamadevan V., Blennow A., Buleon A., Bertoft E. (2018). Distinct properties and structures among B-crystalline starch granules. Starch/Stärke.

[39] Siddiqui S., Ahmed N., Phogat N., Emeje M.O., Blumenberg M. (2022). Varieties, Storage Treatments and Conditions of Tubers. Starch—Evolution and Recent Advances.

[40] Bershtein V.A., Egorov V.M., Kempt T.J. (1994). Differential Scanning Calorimetry of Polymers. Physics, Chemistry, Analysis.

[41] Singh N., Inouchi N., Nishinari K. (2006). Structural, thermal and viscoelastic characteristics of starches separated from normal, sugary and waxy maize. Food Hydrocoll..

[42] Wasserman L.A., Papakhin A.A., Borodina Z.M., Krivandin A.V., Sergeev A.I., Tarasov V.F. (2019). Some physico-chemical and thermodynamic characteristics of maize starches hydrolyzed by glucoamylase. Carbohydr. Polym..

[43] Jenkins P.J., Donald A.M. (1995). The influence of amylose on starch granule structure. Int. J. Biol. Macromol..

[44] Vermeylen R., Goderis B., Delcour J.A. (2006). An X-ray study of hydrothermally treated potato starch. Carbohydr. Polym..

[45] Kozlov S.S., Blennow A., Krivandin A.V., Yuryev V.P. (2007). Structural and thermodynamic properties of starches extracted from GBSS and GWD suppressed potato lines. Int. J. Biol. Macromol..

[46] Noda T., Isono N., Krivandin A.V., Shatalova O.V., Błaszczak W., Yuryev V.P. (2009). Origin of defects in assembled supramolecular structures of sweet potato starches with different amylopectin chain-length distribution. Carbohydr. Polym..

[47] Protserov V.A., Wasserman L.A., Tester R.F., Debon S.J.J., Ezernitskaja M.G., Yuryev V.P. (2002). Thermodynamic and structural properties of starches extracted from potatoes grown at different environmental temperatures. Carbohydr. Polym..

[48] Wunderlich B. (1976). Crystal nucleation, growth, annealing. Macromolecular Physics.

[49] Wasserman L.A., Eiges N.S., Koltysheva G.L., Andreev N.R., Karpov V.G., Yuryev V.P. (2001). The application of different thermodynamic approaches for description structural features in wheat and rye starches. Starch/Stärke.

[50] Whittam M.A., Noel T.R., Ring S.G. (1990). Melting behaviour of A- and B-type starches. Int. J. Biol. Macromol..

[51] Jenkins P.J., Cameron R.E., Donald A.M. (1993). A universal feature in the structure of starch granules from different botanical sources. Starch/Stärke.

[52] Almeida R.L.J., dos Santos Pereira T., de Andrade Freire V., Santiago Â.M., Oliveira H.M.L., de Sousa Conrado L., de Gusmão R.P. (2019). Influence of enzymatic hydrolysis on the properties of red rice starch. Int. J. Biol. Macromol..

[53] Blazek J., Gilbert E.P. (2010). Effect of enzymatic hydrolysis on native starch granule structure. Biomacromolecules.

[54] Langenaeken N.A., De Schepper C.F., De Schutter D.P., Courtin C.M. (2019). Different gelatinization characteristics of small and large barley starch granules impact their enzymatic hydrolysis and sugar production during mashing. Food Chem..

[55] Zou J., Xu M., Wen L., Yang B. (2020). Structure and physicochemical properties of native starch and resistant starch in Chinese yam (*Dioscorea opposita* Thunb.). Carbohydr. Polym..

[56] Han X., Tohge T., Lalor P., Dockery P., Devaney N., Esteves-Ferreira A.A., Fernie A.R., Sulpice R. (2017). Phytochrome A and B regulate primary metabolism in Arabidopsis leaves in response to light. Front. Plant Sci..

[57] Schittenhelm S., Menge-Hartmann U., Oldenburg E. (2004). Photosynthesis, carbohydrate metabolism, and yield of phytochrome-B-overexpressing potatoes under different light regimes. Crop Sci..

[58] Thiele A., Herold M., Lenk J., Quail P., Gatz C. (1999). Heterologous expression of Arabidopsis phytochrome B in transgenic potato influences photosynthetic performance and tuber development. Plant Physiol..

[59] Richter M., Augustat S., Schierbaum F., Richter M., Augustat S., Schierbaum F. (1968). Isolation, characterization and analysis of starch. Selected Methods in Starch Chemistry.

[60] Krivandin A.V., Solov’eva A.B., Glagolev N.N., Shatalova O.V., Kotova S.L. (2003). Structure alterations of perfluorinated sulfocationic membranes under the action of ethylene glycol (SAXS and WAXS studies). Polymer.

[61] Andreev N.R., Kalistratova E.N., Wasserman L.A., Yuryev V.P. (1999). The influence of heating rate and annealing on the melting thermodynamic parameters of some cereal starches in excess water. Starch/Stärke.

[62] Danilenko A.N., Shlikova Y.V., Yuryev V.P. (1994). Equilibrium and co-operative unit of the melting process of native starches with different packing of the macromolecule chains in the crystallites. Biophysics.

[63] Matveev Y.I., van Soest J.J.G., Nieman C., Wasserman L.A., Protserov V.A., Ezernitskaja M.G., Yuryev V.P. (2001). The relationship between thermodynamic and structural properties of low and high amylose maize starches. Carbohydr. Polym..

[64] Bocharnikova I.I., Wasserman L.A., Krivandin A.V., Fornal J., Błaszczak W., Chernykh V.Y., Schiraldi A., Yuryev V.P. (2003). Structure and thermodynamic melting parameters of wheat starches with different amylose content. J. Therm. Anal. Calorim..

[65] Privalov P.L., Potekhin S.A. (1986). Scanning microcalorimetry in studying temperature-induced changes in proteins. Methods Enzymol..

[66] Gernat C., Radosta S., Anger H., Damaschun G. (1993). Crystalline parts of three different conformations detected in native and enzymatically degraded starches. Starch/Stärke.

[67] Mitsui T., Itoh K., Hori H., Ito H. (2010). Biosynthesis and degradation of starch. Bullet. Facul. Agric. Niigata Univ..

[68] Lloyd J.R., Kötting O. (2016). Starch Biosynthesis and Degradation in Plants.

[69] Van Harsselaar J.K., Lorenz J., Senning M., Sonnewald U., Sonnewald S. (2017). Genome-wide analysis of starch; metabolism genes in potato (*Solanum tuberosum* L.). BMC Genom..

