# Supplementary material for: Changes in Structural and Thermodynamic Properties of Starch during Potato Tuber Dormancy
Source: Int J Mol Sci. 2023 May 7;24(9):8397. doi: 10.3390/ijms24098397 (PMC10179465; doi:10.3390/ijms24098397)
Supplement: Supplementary file 1 [file ijms-24-08397-s001.zip › ijms-2375531-SI.pdf]

Supplementary data, ms LA Wasserman et al.

Table S1. Potato genes/proteins presumably involved in starch biosynthesis and degradation, exemplified by RH cultivar

Gene name	Gene symbol RH	Location	Protein RH	Amino acids
StAGP-L	LOC102582750<br>LOC102600909	chr01:63686918..63691316<br>chr07:28459731..28465251	Soltu.DM.01G024440.1<br>Soltu.DM.07G010140.1/40.2	516<br>522/520
StAGP-S	LOC102598180<br>LOC102577790<br>LOC102592916	chr08:8843995..8847025<br>chr07:52424940..52430371<br>chr12:58423902..58431045	Soltu.DM.08G006240.1<br>Soltu.DM.07G022290.1<br>Soltu.DM.12G028820.1	522<br>451<br>590
StPGI1	LOC102585856	chr04:62172117..62197558	Soltu.DM.04G030730.1	1844
StPGM2	LOC102579912<br>LOC102599849	chr04:24544910..24553865<br>chr05:20203158..20205773	Soltu.DM.04G015010.1<br>Soltu.DM.05G013630.1	445<br>353
StGBSS2	LOC102583115	chr02:43711582..43718240	Soltu.DM.02G031690.1	768
StSS3	LOC102577674	chr02:34367685..34383334	Soltu.DM.02G020170.1/70.2	1189/1231
StSS4		chr02:28677632..28687809	Soltu.DM.02G014060.1	993
StGBSS1		chr08:59143802..59147539	Soltu.DM.08G030230.4/30.1	491/608
StSBE1	LOC102603708	chr07:55936360..55953226<br>chr07:55386291..55405743	Soltu.DM.07G026510.2<br>Soltu.DM.07G025710.1	903<br>508
StSBE2	LOC102590711	chr09:3535897..3555467	Soltu.DM.09G004100.1/00.2	859/754
StISA1	LOC102577466	chr07:7354468..7370232	Soltu.DM.07G005540.1/40.2	794/615
StISA3	LOC102577824	chr06:700272..715327	Soltu.DM.06G000410.2/10.3/<br>10.4/10.1	457/376/<br>259/376
StGWD		chr05:9901254..9916669	Soltu.DM.05G009520.2/20.1	1465
StPWD		chr09:66767298..66779235	Soltu.DM.09G030970.2/70.01	921/1203
StAMY1	LOC102598863<br>LOC102582328	chr04:65328723..65330894<br>chr03:35837208..35840386	Soltu.DM.04G033700.1<br>Soltu.DM.03G013410.1/10.2	1203<br>406/372
StAMY2		chr04:68257646..68262571	Soltu.DM.04G037250.2/50.3	408
StAMY3	LOC102599630	chr05:6037245..6053915	Soltu.DM.05G006330.1	1333
StAMY23		chr04:68257646..68262571	Soltu.DM.04G037250.1	403
StLDA	LOC102581262	chr11:4596994..4611918	Soltu.DM.11G004600.1	1333
StLSF1	LOC102603794	chr12:32942100..32949965	Soltu.DM.12G016610.2/10.1	584/479
StBAM1	LOC102577806	chr08:1618564..1622209	Soltu.DM.08G001120.1	542
StBAM5	LOC102584563	chr07:48609120..48613209	Soltu.DM.07G018100.1/00.2	579/
StBAM7	LOC102593066	chr01:73206342..73213592	Soltu.DM.01G033560.1	696
StBAM9	LOC102590483	chr01:60987037..60990400	Soltu.DM.01G022570.1/70.2	461/451
